# Comparative analysis of airborne bacteria and fungi in two salt mines in Poland

**DOI:** 10.1007/s10453-017-9502-6

**Published:** 2017-11-17

**Authors:** Elżbieta Gębarowska, Wojciech Pusz, Jolanta Kucińska, Włodzimierz Kita

**Affiliations:** 1 Division of Agricultural Microbiology, Department of Plant Protection, Wrocław University of Environmental and Life Sciences, Grunwaldzka Str. 53, 50-357 Wrocław, Poland; 2 Division of Phytopathology and Mycology, Department of Plant Protection, Wroclaw University of Environmental and Life Sciences, Grunwaldzki Sq. 24a, 50-363 Wrocław, Poland

**Keywords:** Airborne bacteria and fungi, Bioaerosol, Salt mine, Miners’ hygiene

## Abstract

The aim of this work was to determine the genera or species composition and the number of colony forming units of airborne bacteria and fungi, respectively, in two salt mines in Poland “Wieliczka” (Lesser Poland) and “Polkowice–Sieroszowice” (Lower Silesia). Both of them are working environments characterized by extreme conditions, and additionally “Wieliczka,” officially placed on the UNESCO World Heritage Sites’ list, plays a role of tourist attraction. There are also some curative chambers located in this mine. Air samples were taken once in December 2015, between 6:00 a.m. and 9:00 a.m. There were nine measurement points located about 200 m underground in “Wieliczka” and six measurement points located in the working shafts about 400 m underground in “Polkowice–Sieroszowice.” The total volume of each air sample was 150 L. Air samples, collected in individual measurement points of both salt mines, were inoculated on two microbiological media: potato dextrose agar and tryptic soy agar using the impact method. We identified 10 and 3 fungal genera in the “Wieliczka” Salt Mine and in “Polkowice–Sieroszowice,” respectively. The most common were fungi of the *Penicillium* genus. In both mines, the Gram-positive bacteria of genus *Micrococcus* were detected most frequently. Among identified microorganisms, there were neither pathogenic fungi nor bacteria. The most prevalent microorganisms detected in indoor air were Gram-positive cocci, which constituted up to 80% of airborne microflora. Our results showed that microorganisms recorded in the air samples are not a threat to workers, tourists or patients. Neither pathogens nor potentially pathogenic microorganisms, listed as BSL-2, BSL-3 or BSL-4, were detected. The microbes identified during our analysis commonly occur in such environments as the soil, water and air. Some of the detected bacteria are component of natural microflora of human skin and mucous membranes, and they can cause only opportunistic infections in individuals depending on their health condition.

## Introduction

The purity of the atmospheric air is one of the basic issues of the evaluation procedure of work space quality. Particular attention should be paid to bioaerosols that are ubiquitous in indoor air and potentially harmful to human health. Although the air is considered to be an inimical environment for the reproduction of microorganisms, they are able to endure there and maintain their infectious skills. Bioaerosol, also referred to as organic dust, is usually composed of pathogenic and non-pathogenic bacteria, viruses, fungal spores, matter of plants and animals as well as their primary and secondary metabolites including the bacterial endotoxins or mycotoxins. They are transmitted through airborne route, droplet route, vector-borne route, by direct contact and less by the oral route. The saprophytic and infectious bioaerosols are considered to worsen hygienic conditions contributing to the human allergy. They may cause a waste of food, construction materials and pharmaceuticals. Bioaerosols are also responsible for transmission of many of human, animal and plant infectious diseases, mycosis, upper respiratory infections, viral diseases, fire blight and many others (Douwes et al. 2003; Górny 2004; Heikkinen et al. 2005; https://oshwiki.eu/wiki/Bioaerosols_and_OSH).

The wide and regular monitoring of an environmental cleanliness is increasingly carried out for early detection of the potential risk factors for the social wellness. Actually, there are a few publications describing local occurrence of the biological factors in such specific work spaces as the underground salt mines are (Frączek and Górny 2011; Frączek and Kozdrój 2013; Frączek et al. 2013; Rdzanek et al. 2015), but there are not legal, generally implemented regulations, which would determine suggested limits of airborne bacteria and fungi in extreme work environments.

The salt mines are characterized by a specific microclimate. Brine aerosols have positive impact on the immune system and promote a treatment of various diseases like the upper respiratory ones, allergies, bronchial asthma and many others. Due to the bacteriostatic effect of the salt, the amount of macro- and microorganisms in salt mines is significantly lower than on the earth surface. The number of microorganisms in the mine air is also determined by a degree of dust in corridors and chambers, the microclimate conditions and the people staying there (Chervinskaya 2003, 2007; Hedman et al. 2006; Czajka et al. 2006; Frączek et al. 2013; d’Obyrn and Rajchel 2014; Rashleigh et al. 2014).

The aim of our study was to evaluate the airborne bacteria and fungi in underground spaces in two salt mine areas in Poland: the “Wieliczka” Salt Mine (Lesser Poland) and the KGHM Polska Miedź S.A. Mine Department “Polkowice–Sieroszowice” (Lower Silesia).

As there are no legal regulations in Poland enabling the performance of reliable evaluation of the microbiological quality of the air, in our research we have based on Directive 2000/54/EC, as well as on the suggested limit values for microorganisms in indoor air published by Polish authors (Górny 2004; Zielińska-Jankiewicz et al. 2005; Górny 2010; https://oshwiki.eu/wiki/Bioaerosols_and_OSH) (Table 1). Actually, the United States Environmental Protection Agency (EPA 1989) does not regulate suggested limits of microbes in indoor air (https://www.epa.gov). Some specific recommendations have been given by various scientific organizations. For example, the recommended maximum limits are: 1000 CFUs/m^3^ for the total number of bioaerosol particles set by the National Institute of Occupational Safety and Health (NIOSH), as well as by the American Conference of Governmental Industrial Hygienists (ACGIH) with the culturable count for total bacteria not to exceed 500 CFUs/m^3^ (Yassin and Almouqatea 2010).

**Table 1:** Limit values for microorganisms in indoor air recommended by team of experts of biological factors (ZECB) in Poland (Górny 2004, 2010)

	Working rooms contaminated by organic dust^a^ (CFU/m^3^)	Living areas and public spaces (CFU/m^3^)
Mesophilic bacteria	1 × 10^5^	5 × 10^3^
Gram-negative bacteria	2 × 10^4^	2 × 10^2^
Thermophilic actinomycetes	2 × 10^4^	2 × 10^2^
Fungi	5 × 10^4^	5 × 10^3^
Microorganisms listed as BSL-3 or BSL-4 (pathogens)	0	0

^a^Suggested values should be two times lower for respirable fraction, i.e., 50,000 CFU/m^3^ for mesophilic bacteria, 10,000 CFU/m^3^ for Gram-negative bacteria, 10,000 CFU/m^3^ for thermophilic actinomycetes, 25,000 CFU/m^3^ for fungi

Basic principles that mainly concern the prevention or reduction of biological contaminants have been recommended by Environmental Health Directorate in Canada (1987), European Economic Community (EEC 1990) and World Health Organization (Bonadonna and Marboni 1994). However, some regulations concerning filamentous fungi have been presented in Report on a World Health Organization meeting in 1988 (WHO 1990).

## Materials and methods

### Description of sampling points

This study was performed in two Polish salt mines with different functions and microclimate conditions (Table 2). The “Wieliczka” Salt Mine, located in Wieliczka city (Lesser Poland), has been producing table salt from thirteenth century until 2007 and now is one of the major tourist attractions in Poland playing a role of a cultural monument, as in 1978 it was officially placed on the UNESCO World Heritage Sites’ list. “Polkowice–Sieroszowice,” located in Polkowice city (Lower Silesia) is a modern industrial salt mine resulting from the merger in 1996 of two existing mines: “Polkowice” and “Sieroszowice.”

**Table 2:** Characteristics of mines in which air samples were collected (presented data come from the date of sampling)

	“Wieliczka” salt mine	“Polkowice–Sieroszowice” mine
Air temperature	Temp. 12–15 °C	37–39 °C
Air humidity	40–60%	15–20%
Average depth	100–200 m	400 m
Number of persons	~ 300–400 persons per day	400 persons on shift

Air samples were collected once—on December 7, 2015 (in “Wieliczka”) and on December 14, 2015 (in “Polkowice–Sieroszowice”), between 6:00 a.m. and 9:00 a.m. The sampling points where located about 200 m underground in “Wieliczka” and in the working shafts about 400 m underground in “Polkowice–Sieroszowice.”

There were 9 sampling points in “Wieliczka” Salt Mine, consecutively numbered: 1 and 2 located in rehabilitation-treatment chambers (Eastern Mountains’ Stable, Chamber, Lake Wessel Chamber), 3–6—located on tourist routes (Chamber Modena, Chapel of St. Kinga, Drozdowice Chamber, Gregory fore-shaft), as well as at the ones (7–9) closed to tourist traffic (Tworzyjanków and Daniłowicz Shaft, Franciszek fore-shaft).

There were 6 sampling points in the industrial salt mine “Polkowice–Sieroszowice”: 1—situated in the processing plant (the place where the minor repair works of the mining machinery are carried out), 2 and 3—located in Working face I and Working face II “Najdek” (the locations where combined mining reaches the solid rock), 4—at the Inflow (the place where an atmospheric air reaches the interior of the corridor), 5—at the Vent (the places where the ventilation ducts are connecting and leaving corridors) and 6—located in Miners’ room (the only social place for personnel). The measurement points, besides Vent (Table 4), were located inside of mines.

### Microbiological analysis of bioaerosols

The volume of 50 L of each mine-air sample (total volume collected in each measurement point was 150 L) was inoculated, in three replicates, on two microbiological media: PDA (Potato Dextrose Agar, Difco) and TSA (Tryptic Soy Agar, bioMérieux SA, France) for the isolation of fungi and bacteria, respectively, using the impact method and the Air Ideal 3P sampler. TSA medium was previously supplemented with 30 μg/mL of nystatin (Polfa, Kraków) to inhibit the growth of molds and yeast. The plates with TSA were incubated in 37 °C for 1 day, and in 22 °C for next 3 days. The samples inoculated on PDA were incubated in 30 °C for 4 days, and in 22 °C for next 4 days. After incubation, the number of visible colonies of bacteria and fungi was counted and a general number of colony forming units (CFU) was calculated for each of this group separately. The bacterial colonies were divided into morphological groups according to the macroscopic observation on TSA medium. The representative colonies from each group were subsequently inoculated on TSA medium using a steak plate method. Finally, the pure cultures were identified morphologically, by Gram and spore staining, as well as biochemically, by catalase and API tests (bioMérieux SA, France).

The fungi, grown on PDA medium, were classified to species using the diagnostic keys based on their macroscopic and microscopic morphology (Germain and Summerbell 1996; Fisher and Cook 1998; Samson et al. 2004).

The concentration of the tested bioaerosol (“*X*”) was presented as the number of colony forming units (CFU) in 1 m^3^ of air. It was determined according to the formula: *X* = (*a* × 1000)/*V*, where “*a*” is the average number of CFU and “*V*” is a volume of the inoculated air samples (50 L for each plate). Finally, they were compared to guidelines of Team of Experts of Biological Factors (ZECB) in Poland (Górny 2004, 2010; https://oshwiki.eu/wiki/Bioaerosols_and_OSH).

## Results

### Microbiological analysis of air in “Wieliczka” salt mine

The concentration of bacteria and fungi and the percentage of individual genera and/or species, identified in air of the “Wieliczka” Salt Mine in the selected measurement points, are presented in Table 3. It varied in the tested sampling points. The number of bacteria and fungi ranged from 60 ± 17 to 2027 ± 40 CFU/m^3^ of air and from 50 ± 10 to 750 ± 107 of CFU/m^3^ of air, respectively. The highest concentration of microorganisms (bacteria and fungi together) was observed in curative chambers (Eastern Mountains’ Stable Chamber and Lake Wessel Chamber). In these measurement points, the bacteria were a dominating group (96 and 81%, respectively). The highest concentration of fungal spores was detected in tourist chambers: Chapel of St. Kinga (750 ± 107 CFU/m^3^) and Chamber of Modena (600 ± 78 CFU/m^3^). The lowest number of both groups of the microorganisms was observed in chambers closed for tourists, like Franciszek fore-shaft, Daniłowicz and Tworzyjanków Shaft. In Daniłowicz Shaft, the total number of bacteria was estimated at 93 ± 31 CFU/m^3^, whereas the concentration of fungal spores in 1 m^3^ was 4 times higher (360 ± 68 CFU/m^3^) in comparison with bacteria.

**Table 3:** Concentrations of microorganisms and the percentage of individual bacteria and fungi groups in air samples of the “Wieliczka” salt mine

Number	1.	2.	3.	4.	5.	6.	7.	8.	9.
Place of sample collection	Eastern mountains’ stable chamber	Lake Wessel chamber	Modena chamber	Chapel of St. Kinga	Drozdowice chamber	Fore-shaft Grzegorz	Tworzyjanków shaft	Daniłowicz shaft	Fore-shaft Franciszek
The concentrations of microorganisms (CFU/m^3^ of air)									
Bacteria and fungi together	2120	1607	1240	1043	490	487	223	453	110
Bacteria	2027 (± 40)	1300 (± 89)	640 (± 87)	293 (± 15)	267 (± 29)	93 (± 21)	147 (± 38)	93 (± 31)	60 (± 17)
Fungi	93 (± 12)	307 (± 46)	600 (± 78)	750 (± 107)	223 (± 13)	393 (± 86)	77 (± 23)	360 (± 68)	50 (± 10)

Percentage of individual bacteria and fungi groups									
All bacteria (%)	96	81	52	28	54	19	66	21	55
*Micrococcus* spp.	< 43	40	26	14	23	6	24	6	19
*Micrococcus luteus*	25	26	18	3	8	< 3	15	1	6
*Staphylococcus* spp.	< 1		2		3				
*Staphylococcus epidermidis*		< 1		1		< 3			
*Staphylococcus equorum*	< 2								
*Staphylococcus hominis*	< 1			< 1					
*Staphylococcus saprophiticus*		1	2	< 1	5				
*Staphylococcus xylosus*		1		< 1					
Other cocci	18	6	4	4	10	< 3	9	< 11	18
Endospore-forming bacilli	< 5	< 4			5	1			
Actinomyces	< 1			3			15		
Gram-negative rods	2	2				4	3	3	12

All fungi (%)	4	19	48	72	46	81	34	79	45
*Alternaria alternata*		2							
*Botrytis cinerea*								< 1	
*Cladosporium cladosporioides*	< 1	< 1			< 6	< 14	< 2	71	
*Epicoccum nigrum*	< 1			< 1					
*Penicillium citrinum*	< 1					< 2	< 5		
*Penicillium commune*	< 1						3		
*Penicillium luteum*							< 2		
*Penicillium melagrinum*		< 5	13	18	< 12	12		< 2	6
*Penicillium notatum*	< 2	8	19	23	8	< 38		5	3
*Penicillium purpurogenum*				< 1			< 2		
*Penicillium vermiculatum*		< 1	< 1						
*Penicillium waksmani*	1	4	< 9	20		15	< 18	< 1	3
*Sclerotinia sclerotiorium*			< 1		21		< 2		
*Scopulariopsis* spp.									3
*Trichoderma hamatum*				4		< 1			30
*Trichoderma viride*			7	6		< 1			
*Ulocladium botrytis*		< 1							
Yeast colonies							3		

(±)—standard deviation

The dominant group of bacteria, in tested samples of the air, were Gram-positive cocci belonging to *Micrococcus* genus. In two curative chambers: Eastern Mountains’ Stable and Lake Wessel these cocci were accounted for approximately 67% of the all identified microorganisms (both bacteria and fungi). The staphylococci were also detected in tested samples of the air; however, their number usually did not exceeded 5%. Among them, there were identified *Staphylococcus saprophiticus* and *S. epidermidis*. In the tested air samples, collected in the “Wieliczka” Salt Mine, 17 species of molds belonging to 9 genera were identified. The most frequent were fungi of the *Penicillium* genus, particularly *P. notatum*, *P. meleagrinum* and *P. waksmani*. The *Penicillium* was dominating genus of mycobiota in Gregory fore-shaft (66%) and Chapel of St. Kinga (61%). However, in Daniłowicz Shaft, which is closed for tourists, the dominating species, accounted for 71% of total number of microorganisms, was *Cladosporium cladosporoides*.

### The microbiological analysis of the air samples in “Polkowice–Sieroszowice” salt mine

The estimated concentration of bacterial and fungal aerosols and the percentage of individual genera and/or species in the tested air samples of salt mine in “Polkowice–Sieroszowice” are presented in Table 4. Usually, it did not varied in the individual measuring points. Bacteria were dominant group (89–100%) of microorganisms. Their concentration ranged from 1147 ± 130 to 3827 ± 227 CFU/m^3^. The highest concentration was observed in corridor of Working face I, in Miners’ room as well as at the Inflow and the Vent. The highest concentration of the fungi (295 ± 64 CFU/m^3^) was recorded at the Inflow. No fungal propagules were isolated from the air samples collected in the processing plant and Working face I.

**Table 4:** Concentrations of microorganisms and the percentage of individual bacteria and fungi groups in air samples of the “Polkowice–Sieroszowice” mine

Number	1.	2.	3.	4.	5.	6.
Place of sample collection	Processing plant	Working face I	Working face “Najdek”	Inflow	Vent	Miners’ room
The concentrations of microorganisms (CFU/m^3^ of air)						
Bacteria and fungi together	1507	3827	1157	2802	2943	3412
Bacteria	1507 (± 157)	3827 (± 227)	1147 (± 130)	2507 (± 146)	2933 (± 445)	3307 (± 215)
Fungi	0 (± 0)	0 (± 0)	10 (± 6)	295 (± 64)	10 (± 6)	105 (± 17)

Percentage of individual bacteria and fungi groups						
All bacteria (%)	100	100	99	89	99.7	97
*Micrococcus* spp.	38	19	< 16	10	14	11
*Micrococcus luteus*	50	71	59	65	68	< 72
*Staphylococcus* spp.		< 1	2	< 1	< 1	< 1
*Staphylococcus epidermidis*		< 1	< 1	< 1	< 1	< 1
*Staphylococcus hominis*			< 1	< 1		
*Staphylococcus saprophiticus*			< 1	< 1	1	< 1
*Staphylococcus xylosus*			< 1		< 1	
Other cocci	9	7	< 16	12	15	11
Endospore-forming bacilli	< 2	< 1				1
Actinomyces		< 1		< 1	< 1	< 1
Gram-negative rods	< 1	< 1	< 3			< 1

All fungi (%)	0	0	1	11	< 1	3
*Aspergillus niger*				< 1		
*Cladosporium cladosporioides*						< 3
*Cladosporium herbarum*				1		< 1
*Penicillium citrinum*			1	< 1		
*Penicillium frequentans*				< 1		
*Penicillium implicatum*				< 1		
*Penicillium lanosocereuleum*				< 7		
*Penicillium purpurogenum*						< 1
*Penicillium velutinum*				< 2		
*Penicillium waksmani*				< 1	< 1	< 1

(±)—standard deviation

The Gram-positive cocci were the dominant group of bacteria, especially from the *Micrococcus* genus ranging from 75 to 90%. There were also detected few (less than 5%) staphylococci. Like in the air of the “Wieliczka” Salt Mine, in these aerosols there were identified the single colonies of the *Actinomyces*, Gram-negative rods and spore-forming bacilli. Apart from bacteria, there were also presented 10 species of molds belonging to 3 genera among which *Penicillium* spp. was dominated and was the most frequently isolated from an air inlet to the mine.

## Discussion

In the literature, little is known about bioaerosol composition in indoor air of the mines. Some of these places are not only workplaces but also touristic attractions, as well as the place where some people are cured (Frączek and Grzyb 2010; Grzyb and Frączek 2010; Frączek and Górny 2011; Frączek et al. 2013). In this research, the microbiological analysis of indoor air in two Polish salt mines (“Wieliczka” and “Polkowice–Sieroszowice”) was performed. It was determined the genera or species composition and the number of colony forming units (CFU) of airborne bacteria and fungi. “Wieliczka” Salt Mine, officially placed on the UNESCO World Heritage Sites’ list, plays a role of tourist attraction visited by hundreds of people per day and there are also some curative chambers. Both mines are working environments in extreme conditions; however, there are not legal regulations that would determine the suggested limits of airborne bacteria and fungi in atypical types of workplaces.

In recent years, the interest in biological infectious agents that occurs in the air of public and working spaces has increased (Kubera et al. 2015; Frączek and Grzyb 2010; Frączek and Górny 2011; Frączek et al. 2013). The fungal spores, as well as the bacteria producing endospores, slime, and pigments are undoubtedly more resistant to unfavorable environmental factors. The microorganisms in indoor air are less exposed to such factors as the temperature fluctuations, atmospheric precipitation, UV radiation which is why they are able to survive longer and their amount does not fluctuate seasonally (winter/summer) as much as it does in outdoor air (Chmiel et al. 2015).

The microbiological components of bioaerosols significantly influence on air quality. The airborne bacteria and fungi can be harmful for human health as they are etiological agents of many infections, autoimmune diseases and allergies. They can reach indoor areas through ventilation systems or by means of passive ventilation. The airborne bacteria and fungi can be harmful for human health as they are etiological agents of many infections, autoimmune diseases and allergies. Almost 10% of people worldwide suffer from fungal allergy (Yassin and Almouqatea 2010). That is why an efficient monitoring of bacteria and fungi’s occurrence in indoor air of diverse environments is a very important issue.

The objects of this study in the “Wieliczka” Salt Mine were the chambers utilized curatively, visited by tourists as well as closed for public. In the salt mine “Polkowice–Sieroszowice,” the air samples were taken from the places of salt mining, ventilation inlet and outlet and miners’ chamber. The culture method, used in this research, has many limitations mentioned by different authors (e.g., Štursa et al. 2009; Vartoukian et al. 2010; Rastogi and Sani 2011) and the most important of them is that it underestimates the real amount of microbes in the tested samples because a vast majority of microorganisms (> 99%) are non-cultivable and they could be detected only by molecular methods. In case of cultivable microbes, the single colonies visible on plates can be clumps of bacterial and fungal cells and the ones with different nutritional and environmental requirements will not grow together (on common medium and growth conditions). However, the limit values for microorganisms in indoor air recommended by Team of Experts of Biological Factors (ZECB) in Poland are presented as CFU/m^3^ of air and the results of this study were compared to these standards.

The bacteria were the dominant group of microbes in the “Polkowice–Sieroszowice” salt mine, mainly *Micrococcus* spp. (up to 90%). The bioaerosols in “Wieliczka” Salt Mine were more diverse and fungi prevailed only in 3 measurement points: Gregory fore-shaft (81%), Daniłowicz Shaft (79%) and Chapel of St. Kinga (72%).

The results of microbiological analysis showed that the concentration of the bacteria and fungi in the air of the both tested mines meets the microbiological standards for air quality proposed by the Team of Experts of Biological Factors (ZECB) (Górny 2004, 2010; https://oshwiki.eu/wiki/Bioaerosols_and_OSH). In the “Wieliczka” Salt Mine, the highest number of microorganisms (2120 CFU/m^3^ of air) was recorded in air sampled in Eastern Mountains’ Stable Chamber designated for curative purpose. In the salt mine “Polkowice–Sieroszowice,” the highest concentration of microorganisms (3827 CFU/m^3^ of air) was observed in salt mining area—Working face I.

The appropriate microbial quality of the air, which was observed in our analysis, may be connected with specific atmospheric conditions in the tested mines. In mid-2011, in the “Polkowice–Sieroszowice” salt mine the project of salt extraction on the level of 1 million tons per year was implemented and this task has already been realized in the middle of 2013 making KGHM Polska Miedź the leader of salt producer in Poland. In 2015 it was extracted there almost 234 thousand tons. The low humidity of the air combined with a high temperature recorded in the salt mining area, as well as a high concentration of the salt dust, significantly slows down an excessive growth of microorganisms. In the tested period, the air temperature in the “Polkowice–Sieroszowice” was 38 ± 1 °C and the air humidity ranged from 15 to 20%. In tested curative chambers of the “Wieliczka” Salt Mine, such as the Lake Wessel and the Eastern Mountains’ Stable, the aerosol contains the ions of chlorine, sodium, magnesium and calcium; the air is characterized by unique bacteriological purity, the constant temperature 10–12 °C and high humidity about 80–90% (d’Obyrn and Rajchel 2014). During our experiments, the average temperature in this mine was 12–15 °C and air humidity ranged from 40 to 60%.

In indoor air, microbes are usually distributed unevenly (Yassin and Almouqatea 2010) and in “Wieliczka” Salt Mine we observed the differences in number of bacteria and fungi between measurement points. It ranged from 60 to 2027 CFU/m^3^ of air (bacteria) and from 50 to 750 of CFU/m^3^ of air (fungi). Bioaerosols in the “Wieliczka” Salt Mine were characterized by greater biodiversity in comparison with the “Polkowice–Sieroszowice” where bacteria were the main microbiological component (about 97% of total isolated microorganisms).

Górny and Dutkiewicz (2002) have cited results of the complex study of indoor air, in which the bioaerosol composition of more than 100 flats in 15 towns, located in Upper Silesia conurbation in Poland, was examined. In the group of Gram-positive bacteria, mainly *Micrococcus*/*Kocuria* spp., *Staphylococcus* spp. (in 100% of the tested flats), *Bacillus* spp. (in 90%) and *Nocardia* spp. (in 33%) were detected. Among Gram-negative bacteria, the species of the *Pseudomonadaceae* family (detected in 80% of the examined flats), were dominated. In this study, the Gram-positive cocci *Micrococcus* spp. dominated in indoor air of both mines. They accounted for 84 and 60% of the total number of microorganisms in “Polkowice–Sieroszowice” and in “Wieliczka,” respectively. They commonly occur in natural environment and colonize the surface of human skin. Usually, they are not pathogenic; however, some of them can cause opportunistic infections in certain groups of patients, like those with immunodeficiency (Greenblatt et al. 2004; Murray et al. 2011). Fraczek et al. (2013), who studied the bioaerosol components at the Bochnia Salt Mine Health Resort, also observed that Gram-positive cocci prevailed in indoor air samples and *Micrococcus* spp. accounted for 72% of bacterial and fungal flora. The percentage of other Gram-positive bacteria, like staphylococci, endospore-forming and non-sporulating rods in their study, was also low and ranged from less than 1–4% for individual groups. It was consistent with our analysis, in which the calculated percentage of these microorganisms in both salt mines was lower than 5%. Among the staphylococci there are important human pathogens, particularly *Staphylococcus aureus*, *S. epidermidis*, *S. haemolyticus* or *S. saprophyticus*, which are etiological factors of the staphylococcal food poisoning, infections of skin and wounds, respiratory and urinary system as well as septicemia resulting from blood system infections (Kloos and Bannerman 1999; Talaro and Talaro 2002; Otto 2009; Peacock 2006; Murray et al. 2011). In this study, the percentage of individual staphylococci in both salt mines did not exceed 5% of the total amount of bacteria and fungi. Among them we detected: *S. epidermidis*, *S. hominis*, *S. saprophyticus* and *S. xylosus.* The percentage of individual staphylococci was similar in the study performed at the Bochnia Salt Mine Health Resort (Frączek et al. 2013), but authors identified 8 species of bacteria from this genus, and they did not detect *S. saprophyticus.* It should be noted that we isolated *S. equorum* in the “Wieliczka” Salt Mine—species that is a natural component of the biota of the healthy horses’ skin and it was also isolated from naturally fermented products, e.g., sausages or cheese (Schleifer et al. 1984; Kloos and Bannerman 1999; Leroy et al. 2009; https://en.wikipedia.org/wiki/Staphylococcus_equorum). It occurred (less than 2%) only in Eastern Mountains’ Stable Chamber—formerly authentic stable, where the horses were working in twentieth century and the last one left the underground in 2002 (Gierlotka 2014). Currently it plays a role of curative space.

In the individual tested air samples of both mines less than 100 CFU/m^3^ of air of Gram-negative bacteria were isolated, while their acceptable number in respirable fraction of this type of working area is 1000 CFU/m^3^ of air (Górny 2010).

As described in the literature, fungi are the dominant group representing up to 40% of total microorganisms detected in bioaerosols. The most common are fungi belonging to *Cladosporium*, *Alternaria*, *Penicillium*, *Fusarium*, *Trichoderma* and *Mucor* genera (Pusz et al. 2013, 2014). The representatives of *Penicillium* and *Aspergillus* genera are the most dominant in indoor air, while outdoor bioaerosols mainly contain *Cladosporium* spp. (Cabral 2010; Visagie et al. 2014). According to the results of microbial analysis of indoor air presented by Górny and Dutkiewicz (2002), fungi of the *Penicillium* and *Aspergillus* genera, as well as yeast, were detected in most of examined flats in Poland (in 97, 62 and 52%, respectively). The general percentage of fungi in this study ranged from 4 to 79% in the “Wieliczka” Salt Mine and from 0 to 11% in the “Polkowice–Sieroszowice” salt mine. Primarily the fungi belonging to *Penicillium* genus were detected. In the “Wieliczka” Salt Mine, we identified 8 species of *Penicillium* spp. and most of them represented *P. notatum*, *P. melagrinum* and *P. waksmani* species*. Aspergillus niger*, isolated in this research, was the only representative of the *Aspergillus* genus and it was detected only in Inflow (< 1% of bacteria and fungi together) in “Polkowice–Sieroszowice” mine. Yeast colonies were observed only in Tworzyjanków Shaft (3% of bacteria and fungi together) located in the “Wieliczka” Salt Mine.

The fungi are one of the most significant groups of inhalant allergens. The strong allergens are spores produced by as *Alternaria* spp., *Cladosporium* spp., *Fusarium* spp., *Mucor* spp. or *Aspergillus* spp. (Pusz et al. 2014). The fungi can also cause superficial and systemic mycoses in immunosuppressed patients. The latter ones affect greater amount of tissues, whole organs or group of organs and the most common are respiratory mycoses (Venarske and deShazo 2002). However, the fungi which were detected in both mines in this study are considered to be safe for human health and some of them are listed as BSL-1 (BioSafety Level-1). Neither pathogenic species *Aspergillus fumigatus* nor toxigenic ones *Stachybotrys atra* were detected. Both species are listed as unacceptable in Exposure guidelines for residential indoor air quality (EHD 1987).

Microorganisms belonging to higher levels of BSL involving microbes that pose moderate hazards to personnel and the environment (BSL-2) or may cause serious or potentially lethal disease (BSL-3 and BSL-4) have not been isolated. Microorganisms identified during the analysis presented in this paper are saprotrophs and their law potential to cause diseases makes them to be a little threat for human health. They commonly occur in soil, water and air. Some of the detected bacteria are component of natural microflora of skin and mucous membranes, and they can cause immunopathogenic reactions in individuals depending on their health condition, genetic predisposition, acquired immunity as well as on time of exposition or the number of introduced microorganisms (Szczuka et al. 2013).

In the tested air the *Actinomyces* have also been recorded. These microorganisms are widespread in soil but they could be detected in air as they are important contamination of working and living environment. Their occurrence, in closed spaces, is connected with increased air humidity (Frączek and Kozdrój 2013). In this research they were detected mainly in the “Wieliczka” Salt Mine and their highest concentration was observed in Tworzyjanków Shaft (15%); however, their percentage in total amount of bacteria was low and accounted for 2%.

The bioaerosol composition in salt mines may promote the osmotolerant microbes like *Micrococcus* spp., *Bacillus* spp. or *Penicillium* spp. (Solomon and Viswalingam 2013; Roohi et al. 2012). In fact, the representatives of the *Micrococcus* genus were the most frequently detected microorganisms in all samples collected in the “Polkowice–Sieroszowice” salt mine (75–90%) as well as in both curative chambers of the “Wieliczka” Salt Mine such as Eastern Mountains’ Stable Chamber (68%) and Lake Wessel (66%). However, the percentage of the endospore-forming bacilli in these mines was negligible ranging from 0 to 2% and from 0 to 5%, respectively. The molds of the *Penicillium* genus were the most frequently isolated fungi in both salt mines and predominating microorganisms in Chapel of St. Kinga (66%) and Fore-shaft Grzegorz (66%) located on touristic route but in the “Polkowice–Sieroszowice” mine its percentage ranged from 0 to 13% of total microbes. The results of the study performed by Gunde-Cimerman et al. (2006) and Marbaniang and Nazareth (2007) showed that not only spores but also mycelium of some species of *Penicillium* are able to survive a prolonged suspension in brines and they are more plentiful in colder environments, so the temperature conditions in the Wieliczka’s curative chambers could be conductive for this group of fungi.

In conclusion, the bacterial and fungal aerosol concentration in the tested air of the both salt mines meets the microbiological standards recommended by the Team of Experts of Biological Factors (ZECB) in Poland (Górny 2004, 2010; https://oshwiki.eu/wiki/Bioaerosols_and_OSH). Both the number and the quality of microorganisms recorded at this time in the air samples are not a threat to workers, tourists or patients. Neither pathogens nor potentially pathogenic microorganisms, listed as BSL-2, BSL-3 or BSL-4, were detected. The microbes identified in our analysis commonly occur in such environments as the soil, water and air. Some of the detected bacteria are component of natural microflora of human skin and mucous membranes, and they can cause only opportunistic infections in individuals depending on their health condition. Finally, it needs to be highlighted that in our comparative analysis only one time sampling was performed and only regular monitoring of bioaerosols would provide more information on the occurrence of microorganisms in these mines.
